# Inhibition of Three Citrus Pathogenic Fungi by Peptide PAF56 Involves Cell Membrane Damage

**DOI:** 10.3390/foods10092031

**Published:** 2021-08-29

**Authors:** Wenjun Wang, Guirong Feng, Xindan Li, Changqing Ruan, Jian Ming, Kaifang Zeng

**Affiliations:** 1 College of Food Science, Southwest University, Chongqing 400715, China; wangwenjun_w@outlook.com (W.W.); fengguirong0128@outlook.com (G.F.); 18227589580@163.com (X.L.); changqing.r@hotmail.com (C.R.); mingjian1972@163.com (J.M.); 2 Research Center of Food Storage & Logistics, Southwest University, Chongqing 400715, China; 3 Key Laboratory of Plant Hormones and Development Regulation of Chongqing, Chongqing 401331, China

**Keywords:** peptide PAF56, citrus fruit, changes cell structure, spores, membrane permeability

## Abstract

The peptide PAF56 (GHRKKWFW) was reported to be an effective control for the main diseases of citrus fruit during postharvest storage. However, the mechanism of action of PAF56 is still unknown. In this paper, PAF56 might not induce defense resistance of citrus fruit. The SEM results visually indicated that the fungi mycelia became shrunken and distorted after being treated with PAF56. The destructive effects of PAF56 on the mycelial cell membrane of three kinds of pathogenic fungi (*Penicillium digitatum*, *Penicillium italicum*, and *Geotrichum citri-aurantii*) were verified by the K^+^ leakage and the release of nucleic acid. Furthermore, the interaction between peptide PAF56 and the pathogen spores was investigated, including the changes in cell membrane permeability and dynamic observation of the interaction of fluorescein labeled TMR-PAF56 and *Geotrichum candidum* spores. The results indicated that the antifungal activity of PAF56 on spores was time-dependent and directly related to the membrane damage. This research provided useful references for further research and practical application of peptides.

## 1. Introduction

Citrus is the type of fruit crop with the highest production worldwide, and the citrus industry has great economic importance. *Penicillium digitatum* (green mold), *P. italicum* (blue mold), and *Geotrichum citri-aurantii* (sour rot) are well known as the predominant citrus pathogens causing postharvest diseases during fruit storing and transportation. Recently, researchers have been trying to use various safe and effective approaches to control these diseases.

Antimicrobial peptides (AMPs) were widely studied as novel antibiotics and have been applied for controlling phytopathogens in agriculture, postharvest conservation, medical industry, and so on. Increasing antimicrobial peptides has been proved to be able to control infectious diseases of fruit and vegetables such as citrus and tomato [1,2,3]. In particular, short-chain cationic antimicrobial peptides attract the attention of researchers due to their cheap synthesis costs and excellent antimicrobial efficiency. Markedly, PAFs were a group of de novo designed hexapeptides with a good controlling effect against plant filamentous fungi [4]. PAF26 (RKKWFW) was reported to be an effective inhibitor for the growth of *P. digitatum* in vivo and in vitro [5,6], without lytic or cytotoxic effects on human cells. Further studies have shown that PAF26 has multiple effects on *P. digitatum* that ultimately result in permeation and killing [7,8]. When the N-terminal of PAF26 was extended by glycine and histidine residues (GH), PAF56 was obtained. It has been proved that PAF56 could control several pathogens, including fungi *P. digitatum*, *Fusarium oxysporum*, and Gram-negative bacterium *Escherichia coli* [9]. In our previous research, PAF56 exhibited an effective control on green and blue mold and sour rot in citrus fruit without a hemolytic effect. PAF56 could change the selective permeability of *P. digitatum*, *P. italicum*, and *G. candidum* mycelia after 48 h treatment, while SG (SYTOX Green) would enter the cell and bind to the nucleic acid, and emitted strong green fluorescence. The extracellular conductivity significantly increased with the increasing concentration of PAF56 [10]. The fungicidal mechanism of the control of PAF56 for the diseases of citrus fruit has not been revealed. It is not clear whether the mechanism of the control of active peptide PAF56 for those diseases is related to induced fruit defense resistance. In addition, fungi initiate their infection by disseminating spores, and then spores swell and germinate into hyphae, which results in severe yield loss in the citrus industry.

In the present study, we studied the effect of PAF56 on spores, and the mechanism related to cell membrane was further explored focusing on two aspects of fungal spores and mycelia.

## 2. Materials and Methods

### 2.1. Synthetic Peptide

Peptides were purchased from GenScript Corporation (Nanjing, China) at >90% purity. PAF56 (GHRKKWFW) was synthesized by the solid-phase method using 9-fluorenylmethoxycarbonyl (Fmoc)-type chemistry. TMR-PAF56 (PAF56 labeled with tetramethyl-rhodamine) modified covalently at its N-terminus was also synthesized. Stock solutions of PAF56 at 1 mmol L^−1^ were prepared in sterile ultrapure water, and stock solutions of TMR-PAF56 at 4 mmol L^−1^ were prepared in 5 mmol L^−1^ 3-(N-morpholino)-propane sulfonic acid (MOPS) and pH 7 buffer and stored in low-light conditions at −40 °C.

### 2.2. Fungal Strains

*P. digitatum*, *P. italicum*, and *G. candidum* were all isolated from the surface of naturally infected citrus fruit and identified by morphology and sequence of the internal transcribed spacer (ITS) rDNA region. The pathogens were purified and cultured in potato dextrose agar (PDA) plate at 25 °C [11]. Spores were obtained from 7-days-old plates and washed with sterile distilled water containing 0.1 g kg^−1^ Tween-80. Spores were titrated with a hemacytometer.

### 2.3. Fruit and Treatment

Citrus fruit [*Citrus sinensis* (L.) Osbeck] were harvested at their commercial maturity from a local orchard (Beibei, Chongqing). After harvesting, the fruit were selected based on uniform size, color, and absence of defects. The fruit were surface-disinfected with 2% (*v*/*v*) sodium hypochlorite for 2 min, washed with water, and air-dried at room temperature (20 °C).

To test whether defense resistance is induced in citrus by PAF56, two holes (3 mm × 4 mm) were drilled at two sites around the equator of each fruit. PAF56 (10 μL, 64 μmol L^−1^) was pipetted into each wound site. Citrus fruit inoculated with ultrapure water only was set as the control. After 24 h, a 10 μL suspension of 1 × 10^4^ CFU mL^−1^ of fungi (*P. italicum*, *P. digitatum*, or *G. candidum)* spores was inoculated into a new rind site that was 1 cm away from the initial treated point. Three replicates (15 fruits per replicate, 2 wounds per fruit) were prepared for each group. All fruits were stored at 25 °C and 90% RH (relative humidity). The disease incidence (DI) and the lesion diameter (LD) were assessed daily. The mean values ± S.D. of DI and LD were calculated [12].

### 2.4. Scanning Electron Microscope (SEM) Analysis

We observed the damage of PAF56 to the mycelia morphology of the fungi by scanning electron microscope (SEM). Mycelia cultured in potato dextrose broth (PDB) liquid for 2 d were collected, washed, and resuspended in the PAF56 solutions (0, 10, and 100 μmol L^−1^) for 48 h. Mycelia morphology of the three fungi was observed according to the previous method with a minor modification [13], by using a JEOL JSM-6510LV SEM (JEOL, Tokyo, Japan) operating at 25 kV.

### 2.5. Measurement of Indicators Related to Change of Permeability of Cell Membrane

The efflux of K^+^ of cytoplasmic components is an important indicator of the increasing permeability of the cell membrane. PAF56 solutions (10 or 100 μmol L^−1^) used in each treatment group were prepared, while controls without PAF56 were treated similarly. The concentration of extracellular potassium in the supernatant and release of cytoplasmic constituents were measured by flame atomic absorption spectroscopy (Shimadzu AA6300, Kyoto, Japan) and using a Multiskan Spectrum microplate spectrophotometer at 260 nm (BioTek Instruments, Inc., Winooski, VT, USA), respectively [12,14,15]. The mycelia were collected after shaking at 25 °C for 2 d and washed before resuspension in sterilized distilled water (for the measurement of extracellular potassium concentration) or phosphate buffer (0.05 mol L^−1^, phosphate, pH 7.0) (for the measurement of the release of cytoplasmic constituents). The peptides were added at concentrations of 10 or 100 μmol L^−1^. The concentration of free K^+^ in the suspensions and absorbance values at 260 nm in the supernatant were measured after treatment at 0, 3, 6, 9, 12, 24, and 48 h. Each treatment was performed in triplicate, and the control lacked peptides.

### 2.6. The Fungicidal Kinetics of PAF56 against Spores

The fungicidal kinetics of PAF56 against *P. digitatum*, *P. italicum*, and *G. candidum* spores were measured as previously described [5,16] with minor modifications. The final concentration of PAF56 was used at 64 μmol L^−1^. Spores (10^3^ CFU mL^−1^) were mixed with PAF56 in sterile distilled water. Group without the use of peptide was the control group. The spore suspension was then incubated at 25 °C. Samples of 50 µL were spread onto PDA plates at each time point after incubation. The CFU was counted after the plates were incubated for 48 h at 25 °C. Treatments were prepared in triplicate.

### 2.7. Damage Effect of PAF56 on Membrane Permeability of Spores by Fluorescence Microscopy

Damage effect of PAF56 on membrane permeability of the three fungi spores was observed by an Eclipse TS100 epifluorescence microscope (Nikon Corporation, Minato City, Japan), and the fluorescent dye SYTOX Green (SG) (Molecular Probes; Invitrogen Corp, Carlsbad, CA, USA) was used in this experiment according to the previous description with minor modifications [10,17]. Aliquots of 450 μL of spores (10^7^ CFU mL^−1^) were incubated in 1.5 mL light-safe microcentrifuge tubes, and subsequently, 50 μL of PAF56 (the final concentration was 64 μmol L^−1^) was added and allowed to grow. Group without the use of peptide was the control group. The suspensions of fungal spores were stained with fluorescent dye SYTOX Green after incubation. Fluorescence was examined and photographed with FITC filter sets at different time points. We also captured the simultaneous brightfield images.

Confocal microscopy was used to observe the effect of PAF56 on spores as well. Since the spores of *G. candidum* are bigger than those of *P. digitatum* and *P. italicum*, it is easier to observe. Thus, this experiment took *G. candidum* as an example. Confocal microscopy was used to observe the distribution of fluorescent-labeled TMR-PAF56 in spores and the damage to cell membrane at different times. Aliquots of 450 μL of spores (10^7^ CFU mL^−1^) were incubated in 1.5 mL light-safe microcentrifuge tubes. Subsequently, 50 μL of TMR-PAF56 (the final concentration was 64 μmol L^−1^) and fluorescent dye SG (the final concentration was 0.2 μmol L^−1^) were added. The suspensions were photographed by an Olympus FV1000 laser confocal microscope (Olympus Corporation, Shinjuku City, Japan) with FITC filter and Rhodamine Red-x sets. Simultaneous brightfield images were also photographed.

## 3. Results

### 3.1. Analysis of PAF56’s Effect on the Induction of Defense Resistance in Citrus Fruit

**Figure 1:**
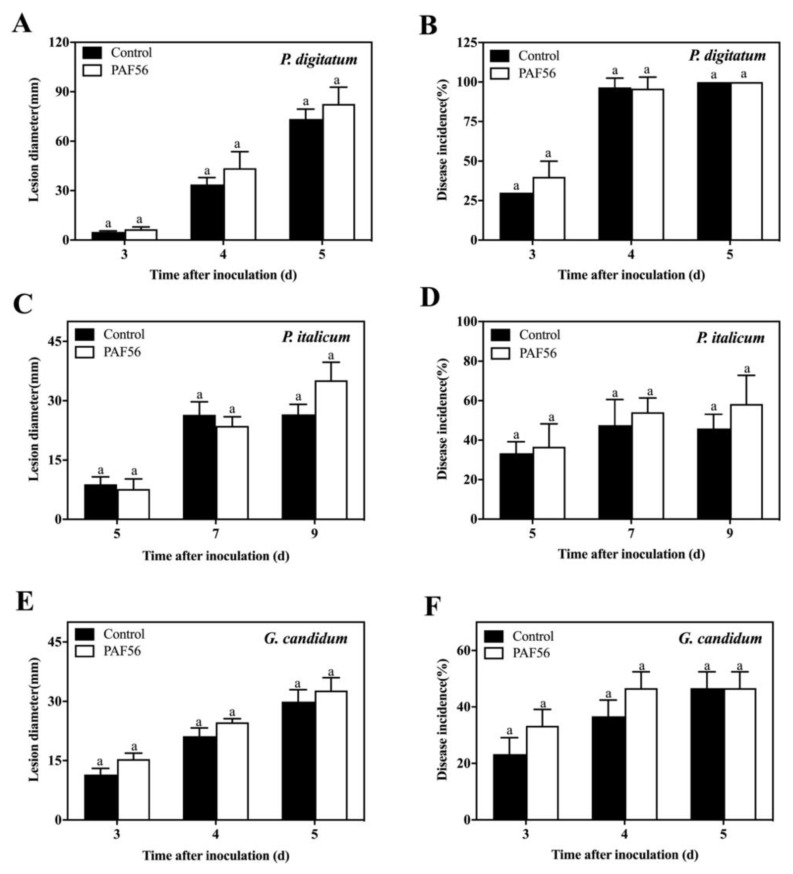
Effect of PAF56 (Inoculation in the different wounds) on lesion diameter and disease incidence of citrus fruit caused by *P. digitatum* (**A**,**B**), *P. italicum* (**C**,**D**), and *G. candidum* (**E**,**F**). Values are mean ± SD. The letters ‘a’ indicate no differences at the 0.05 level. The analysis was conducted using the data from the same pathogen on the same day.

The fruit test experiment was carried out to judge whether PAF56 could induce the defense resistance of the citrus fruit by inoculating PAF56 into the citrus fruit in the different wounds (Figure 1). The disease incidence and lesion diameter results show that there were no significant difference between the citrus fruit inoculated with PAF56 and the control group inoculated with sterile distilled water during the whole period of the disease development. The results for the three kinds of pathogenic fungi, *P. italicum*, *P. digitatum*, and *G. candidum*, were similar, which demonstrates that PAF56 might not induce defense resistance of citrus fruit.

### 3.2. Morphological Alterations of Fungal Mycelia in Response to PAF56

**Figure 2:**
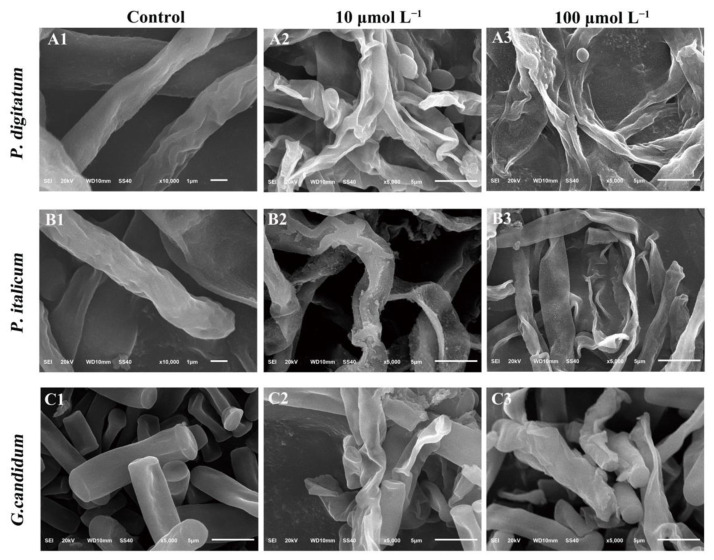
SEM images of *P. digitatum* (panels **A**)*, P. italicum* (panels **B**), and *G. candidum* (panels **C**) mycelia treated with PAF56. Mycelia were incubated in 5% PDB without PAF56 (**A1**–**C1**) or with PAF56 at final concentrations of 10 μmol L^−1^ (**A2**–**C2**) or 100 μmol L^−1^ (**A3**–**C3**).

The effect of PAF56 on the morphology of the three fungi mycelia was examined with SEM (Figure 2). It could be observed that the mycelial morphology of the two concentrations of the treated groups changed considerably compared with the control group. The mycelia treated with PAF56 of 10 or 100 μmol L^−1^ became deformed, shrunken, and distorted, and higher concentrations showed higher damage. By comparison, the surface morphology of *P. digitatum* hyphae changed most obviously after being treated by PAF56 at 100 μmol L^−1^. Meanwhile, the mycelia were seriously collapsed, distorted, and fibrous.

### 3.3. Effect of PAF56 on the Efflux of K^+^ and the Release of Cytoplasmic Constituents of Mycelia

**Figure 3:**
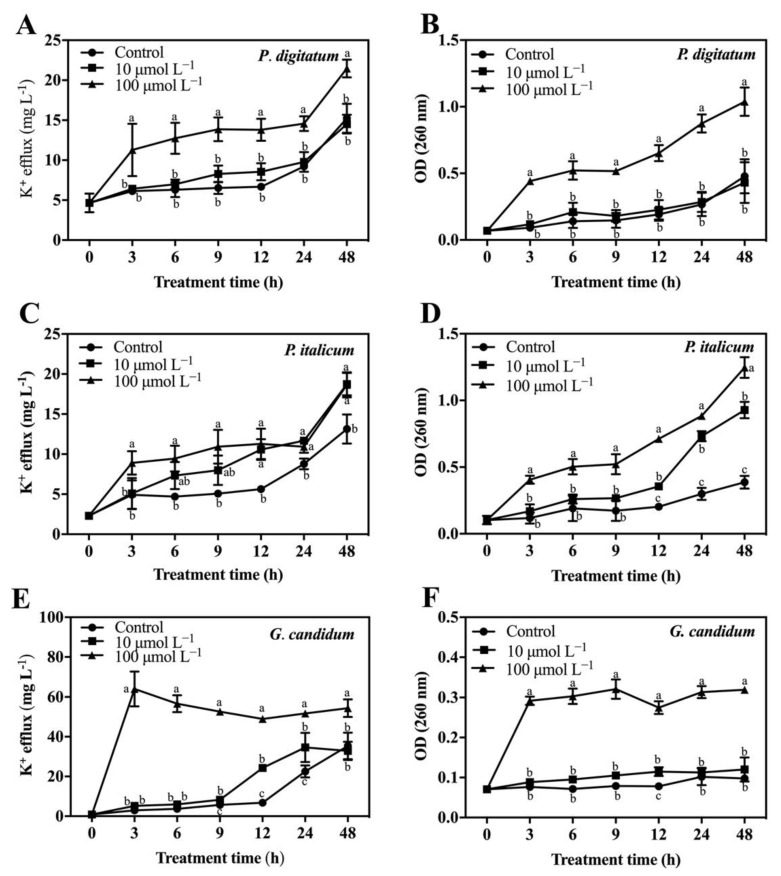
K^+^ efflux and release of cellular constituents of *P. digitatum* (**A**,**B**), *P. italicum* (**C**,**D**), and *G. candidum* (**E**,**F**) mycelia treated with PAF56. Mycelia were incubated in 10 μmol L^−1^ or 100 μmol L^−1^ or without PAF56 (control) solutions. The mycelia were washed before resuspension in sterilized distilled water (for the measurement of extracellular potassium concentration) or phosphate buffer (0.05 mol L^−1^, phosphate, pH 7.0) (for the measurement of the release of cytoplasmic constituents). The concentration of free K^+^ in the suspensions without mycelia was measured by flame atomic absorption spectroscopy, and the release of cytoplasmic constituents was measured using a Multiskan Spectrum microplate spectrophotometer at 260 nm. Vertical bars indicate the standard error of the means. The letters ‘a’, ‘b’, and ‘c’ indicate significant differences at the 0.05 level.

The potassium ions (K^+^) leakage of mycelia was caused by PAF56 treatment (Figure 3A,C,E). As shown, the concentration of extracellular K^+^ in the group treated with PAF56 and the control group increased gradually during the measurements, and PAF56 treatment could significantly induce the efflux of K^+^. The K^+^ leakage of the PAF56 group with the concentration of 100 μmol L^−1^ was significantly higher (*p* < 0.05) than that of the control (no peptide) group. Specifically, *G. candidum* mycelia was seriously damaged at high concentration (100 μmol L^−1^), which led to the leakage of K^+^. After the treatment with peptides, the concentration of the extracellular K^+^ could be reached at 3 h (Figure 3E). This may be related to the characteristics of *G. candidum*, the cell membrane of which is easier for PAF56 to break.

The release of cytoplasmic constituents from the treated mycelia of the three fungi was measured as well. To analyze the leakage of cytoplasmic constituents in the cells of these pathogenic fungi mycelia after being treated with PAF56, the OD_260_ value was measured. The results show that the OD_260_ value of the PAF56 treatment group revealed an increasing release of cytoplasmic constituents with the exposure time increasing (Figure 3B,D,F), compared with that of the control group. This means that the release of cytoplasmic constituents results from these pathogenic fungi mycelia being treated with PAF56.

### 3.4. Effect of PAF56 Treatment on the Membrane Permeability of Fungal Spores

**Figure 4:**
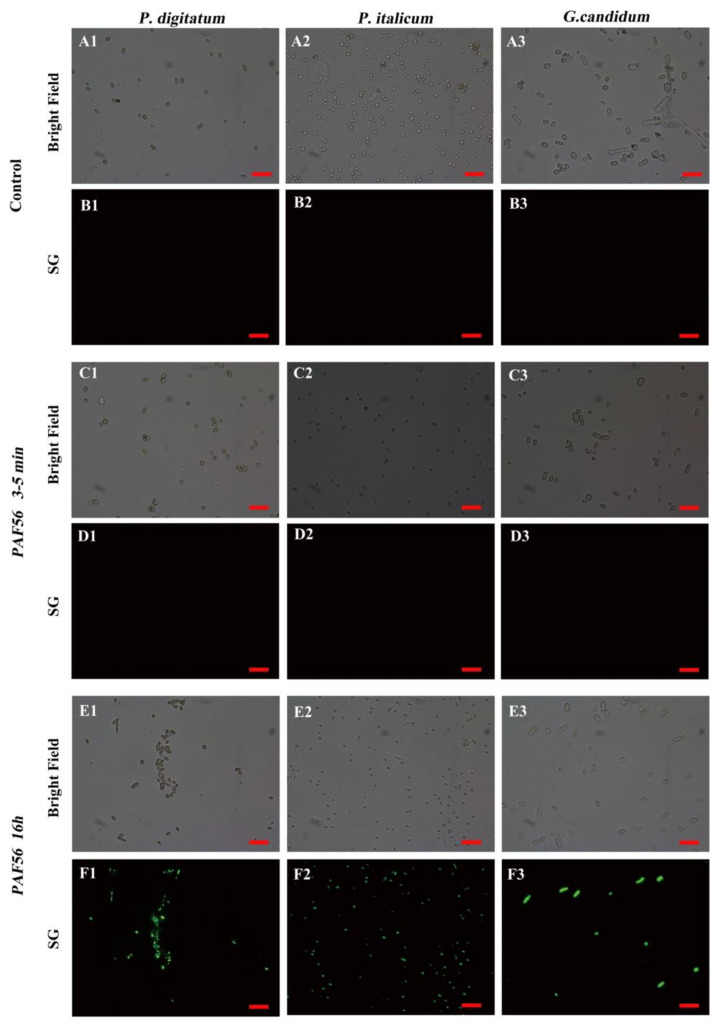
Effect of PAF56 treatment on the membrane permeability of *P. digitatum* (**A1**–**F1**), *P. italicum* (**A2**–**F2**), and *G. candidum* (**A3**–**F3**) spores (bars = 20 μm). Spores were incubated in light-safe microcentrifuge tubes, and subsequently, PAF56 (the final concentration was 64 μmol L^−1^) was added. Group without the use of peptide was the control group. The suspensions of fungal spores were stained with fluorescent dye SYTOX Green (SG) after incubation, and then fluorescence was examined and photographed at different time points.

The effects of PAF56 treatment on the membranes of the three fungi spores were observed by SG fluorescent staining and fluorescence microscopy. Figure 4A,C,E are the images of the bright field of Figure 4B,D,F. The results show that no spores in the control group could emit green SG fluorescence (Figure 4B1–B3). PAF56 could not induce the spores to emit green SG fluorescence in a short time (3–5 min) (Figure 4D1–D3) either. However, PAF56 treatment after 16 h could cause the emitting of green SG fluorescence for all spores (Figure 4F1–F3). To sum up, PAF56 treatment could change the spore membrane permeability of *P. digitatum*, *P. italicum*, and *G. candidum*, allowing SG to enter the spores and bind with the nucleic acid. These results are probably related to the duration of the treatment with PAF56.

### 3.5. Time-Kill Kinetics of PAF56 against Fungal Spores

**Figure 5:**
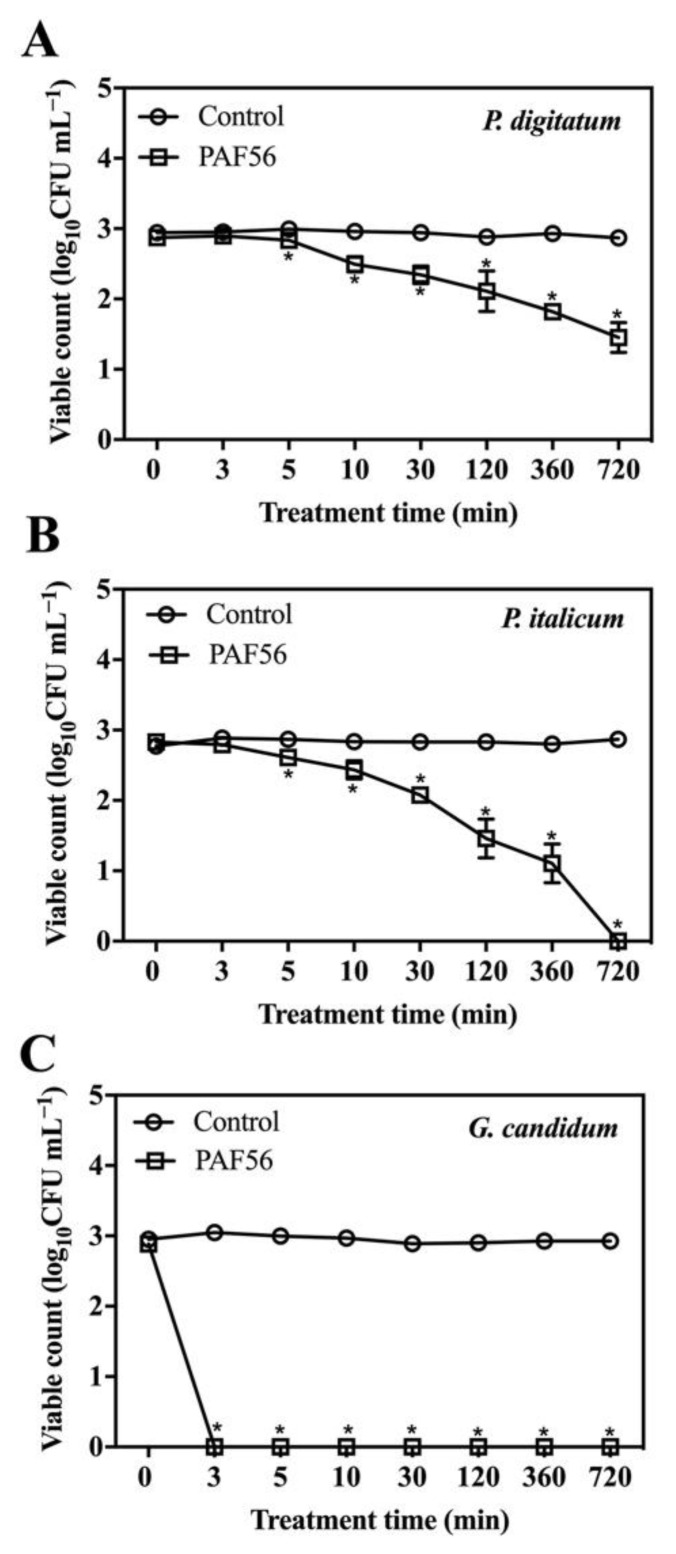
Time–kill kinetics of peptides PAF56 against *P. digitatum* (**A**), *P. italicum* (**B**), and *G. candidum* (**C**) spores. Spores (10^3^ CFU mL^−1^) were mixed with PAF56 (64 μmol L^−1^) in sterile distilled water. Group without the use of peptide was the control group. Samples of 50 µL were spread onto PDA plates at each time point after incubation. The CFU was counted after the plates were incubated for 48 h at 25 °C. Vertical bars indicate the standard error of the means. The mark * represents the significant differences (*p* < 0.05) between PAF56 and the control group.

PAF56 was incubated with the three pathogenic fungi, and the time–kill kinetics curves of PAF56 were plotted at different times by measuring the number of colonies (Figure 5). Compared with the control group, the CFU number of *P. digitatum*, *P. italicumi*, and *G. candidum* decreased with incubation time after adding PAF56 to all treatment groups. The effect of PAF56 on *P. digitatum* and *P. italicum* spores was significantly related to the action time. The longer the action time was, the lower the detected CFU number. The effect of PAF56 on *G. candidum* spores was very strong in a short treatment time. The number of colonies of *G. candidum* was 0 at 3 min. This might be related to the differences in the structure of spores of the three pathogens and their sensitivity to PAF56.

### 3.6. Time-Lapse Confocal Fluorescence Microscopy Analyses of the Interaction of TMR-PAF56

**Figure 6:**
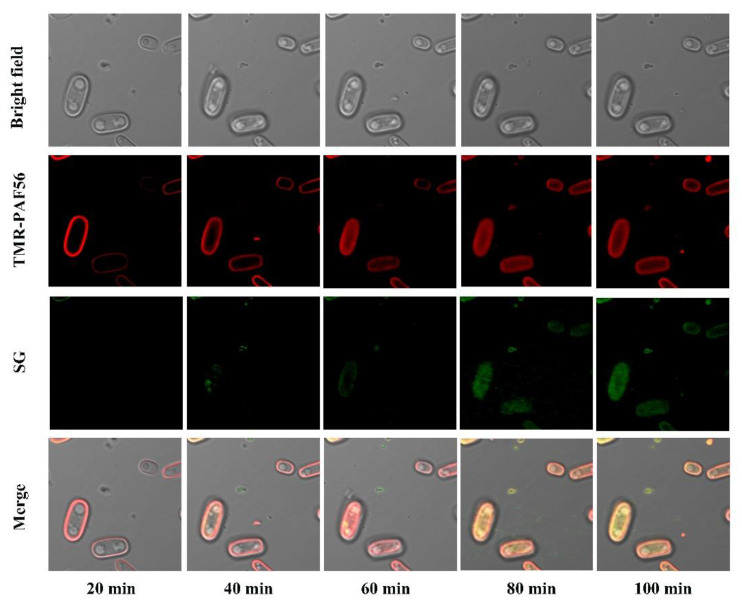
Time-lapse confocal fluorescence microscopy analyses of the interaction of TMR-PAF56. Spores were incubated in light-safe microcentrifuge tubes. Subsequently, TMR-PAF56 and fluorescent dye SG were added. The suspensions were photographed by an Olympus FV1000 laser confocal microscope with FITC filter and Rhodamine Red-x sets at different time points. Simultaneous brightfield images were also photographed.

As presented in Figure 6, TMR-PAF56 could emit red fluorescence after excitation, and the red fluorescence first appeared on the spore surface of *G. candidum*. Then, the red fluorescence was detected in the spores, which indicated that TMR-PAF56 firstly gathered on the surfaces of the spores, then slowly entered the spores as the time extended, and finally spread to the insides of the whole spores. At the same time, the green fluorescence of SG was detected. The green SG fluorescence was not observed at the beginning of 20 min but appeared at the following time points. Almost all spores produced a strong green SG fluorescence at 100 min, which indicated TMR-PAF56 destroyed the spore membrane. The results were that SG entered the spores, bound with cytoplasmic constituents, and emitted green SG fluorescence. The appearance and increase of green SG fluorescence corresponded to the degree of damage of TMR-PAF56 to the cell membrane. In summary, the damage degree of TMR-PAF56 to the membrane of *G. candidum* spore is closely related to the acting time, and the main target of TMR-PAF56 may be the membrane of spores.

## 4. Discussion

It is important to study the mechanism of peptides’ effects on pathogenic fungi for their better application in the future. As a safe peptide without hemolysis [10], it is necessary to conduct in-depth research on PAF56.

Current studies on the controlling of fruit disease have found that a few substances can not only directly inhibit fungi, but also induce fruit resistance to disease. The incidence of disease was significantly reduced, such as antagonistic yeast [18], salicylic acid [19], and chitosan oligosaccharide [20,21,22]. However, few studies have reported whether the peptides can induce fruit resistance, while only some researchers thought that the peptides could stimulate the immune response and function in vivo [23,24]. Therefore, in this study, PAF56 and pathogenic spores were inoculated in different holes of citrus fruit to verify whether PAF56 could induce fruit resistance. The results show that PAF56 did not induce disease resistance in citrus fruit (Figure 1). This indicated that the control of green and blue mold and acid rot of citrus fruit was due to the direct action of PAF56 on pathogenic fungi. Therefore, we further investigated the mechanism of the peptide PAF56 on pathogenic fungi in vitro.

The cell membrane represents the first and last line of defense for ensuring the normal function and ultimately the viability of the cell. The surface morphological changes of mycelia treated with PAF56 were observed by scanning electron microscope. The results show that PAF56 caused mycelia wrinkles, irregular distortion, and serious morphology changes in *P. digitatum*, *P. italicum*, and *G. candidum* after 16 h inoculation (Figure 2). Those results were similar to those found with Buforin 2, some essential oils, and citral [12,25,26].

Previous studies showed that the value of the extracellular conductivity of the three fungi would increase. We further measured the leakage of K^+^ and the release of cytoplasmic constituents of fungal mycelia after treatment with PAF56 (Figure 3). These three indicators are often used to assess irreversible damage to cell membranes and cytoplasm. The experimental results further confirm that PAF56 could increase the permeability of the cell membranes of the three fungi.

*P. digitatum*, *P. italicum*, and *G. candidum* initiate their infection of citrus fruit by disseminating spores. It is extremely important to control spore germination and infection. The destruction of spores’ membranes in *P. digitatum*, *P. italicum*, and *G. candidum* treated with PAF56 over short term (3–5 min) and long term (16 h) was observed by SG fluorescence staining technology and fluorescence microscope (Figure 4). The results show that the spore membrane of these three pathogenic fungi could be destroyed after treatment with PAF56 for 16 h. It is noteworthy that the treatment time of 3–5 min is insufficient to destroy the spore membrane of the three pathogens. Furthermore, the kinetic curves of spores killed by PAF56 were plotted (Figure 5). It is also noteworthy that the effect of PAF56 on *P. digitatum* and *P. italicum* spores became more and more remarkable with the prolongation of time, and the kinetic curves were almost linear, indicating that the effect of PAF56 on *P. digitatum* and *P. italicum* spores was positively correlated with treatment time. The difference is that the effect of PAF56 on *G. candidum* spores was very significant in a short period of time, which might be related to the difference in the structure of spores of pathogenic fungus and the differing sensitivity of spores to PAF56. Then, PAF56 was labeled by fluorescence labeling, and the localization of TMR-PAF56 in *G. candidum* spores was observed by laser confocal microscopy at different times (Figure 6). The results show that the damage degree of TMR-PAF56 on *G. candidum* spores was closely related to the action time, which is similar to previous studies [27,28]. Fluorescent labeling of PAF56 might lead to a decrease in its antifungal activity. The effect of PAF56 on spores was directly related to the destruction of the membrane.

## 5. Conclusions

In summary, PAF56 treatment could destroy the cell structure of *P. digitatum*, *P. italicum*, and *G. candidum* mycelia, change their permeability, and cause leakage of their contents. Citrus fruit could not be induced by PAF56 to produce disease resistance. The effect of PAF56 on spores is directly related to the breaking of cell membranes and the acting time. The results of this study will provide a useful reference for related studies and applications in citrus production and storage.

## Data Availability

Not applicable.
